# Draft genome sequences and description of *Lactobacillus rhamnosus* strains L31, L34, and L35

**DOI:** 10.4056/sigs.5048907

**Published:** 2014-02-15

**Authors:** Prapaporn Boonma, Jennifer K. Spinler, Xiang Qin, Chutima Jittaprasatsin, Donna M. Muzny, Harsha Doddapaneni, Richard Gibbs, Joe Petrosino, Somying Tumwasorn, James Versalovic

**Affiliations:** 1 Interdisciplinary Program of Medical Microbiology, Graduate School, Chulalongkorn University, Bangkok, Thailand; 2 Texas Children’s Microbiome Center, Department of Pathology, Texas Children's Hospital, Houston, Texas, USA; 3 Department of Pathology & Immunology, Baylor College of Medicine, Houston, Texas, USA; 4 Department of Molecular Virology and Microbiology, Baylor College of Medicine, Houston, Texas, USA; 5 Human Genome Sequencing Center, Baylor College of Medicine, Houston, Texas, USA; 6 Department of Microbiology, Faculty of Medicine, Chulalongkorn University, Bangkok, Thailand

**Keywords:** *Lactobacillus rhamnosus*, comparative genomics, probiotics, lactic acid bacteria, anti-inflammatory

## Abstract

*Lactobacillus rhamnosus* is a facultative, lactic acid bacterium in the phylum *Firmicutes*. *Lactobacillus* spp. are generally considered beneficial, and specific strains of *L. rhamnosus* are validated probiotics. We describe the draft genomes of three *L. rhamnosus* strains (L31, L34, and L35) isolated from the feces of Thai breastfed infants, which exhibit anti-inflammatory properties *in vitro*. The three genomes range between 2.8 – 2.9 Mb, and contain approximately 2,700 protein coding genes.

## Introduction

*Lactobacillus* is the largest of three genera within the family *Lactobacillaceae*, and belongs to one of the dominant phyla, *Firmicutes*, in the human microbiome [1]. *Lactobacillus* spp. are naturally isolated from fermented foods [2], and are key members of the human microbiota, reviewed in [3]. In humans, they colonize the oral cavity, gastrointestinal and urogenital tracts, and breast milk [4]. As a whole, this genus is beneficial to humans, possesses many probiotic traits, and is rarely associated with disease.

The human-intestinal isolate, *L. rhamnosus* strain GG, is one of the most studied and applied probiotics. Research has shown that *L. rhamnosus* GG can modulate host immunity *in vitro* by decreasing inflammatory cytokine production from various eukaryotic cell lines [5,6], induces intestinal mucin gene expression subsequently inhibiting pathogen adherence *in vitro* [7]; and attenuates *in vitro* barrier dysfunction induced by inflammatory cytokines [8]. Here we present the draft genomes and classification summary of three potential probiotic *L. rhamnosus* strains L31, L34, and L35 isolated from the feces of Thai breastfed infants [9]. Genome sequencing and comparisons of L31, L34, and L35 with the species type-strain, *L. rhamnosus* GG should help researchers identify distinguishing genetic features important for specific probiotic traits.

## Classification and features

**Figure 1:**
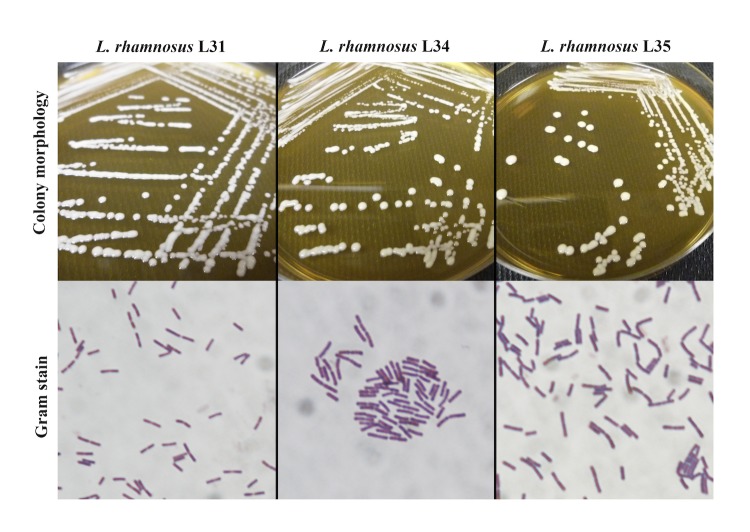
The phylogenetic tree represents the relationships of *L. rhamnosus* strains L31, L34, and L35 with respect to several members of the genus *Lactobacillus*. The strains and their corresponding GenBank accession numbers for 16S rRNA genes are: *L. rhamnsosus* strain GG, NC_013198, *L. salivarius* strain CECT 5713, NC_017481, *L. ruminis* strain ATCC 27782, NC_015975, *L. reuteri* strain JCM 1112, NC_010609, *L. fermentum* strain CECT 5716, NC_017465, *L. johnsonii* strain NCC 533, NC_005362, *L. delbrueckii subsp. bulgaricus* strain ATCC 11842, NC_008054, *L. acidophilus* strain NCFM, NC_006814. Full-length 16S rRNA gene sequences were aligned using ClustalW, and phylogenetic inferences were obtained using the maximum-likelihood method within the MEGA 5.2 software [11] with 1,000 bootstraps. *B. subtilis* strain 6051 HGW (NC_020507) was used as an outgroup.

Within the phylum *Firmicutes*, the family *Lactobacillaceae* contains three genera: *Lactobacillus, Paralactobacillus,* and *Pediococcus*; *Lactobacillus* being the largest with latest estimates ranging between 227-230 species (http://www.dsmz.de/bacterial-diversity/prokaryotic-nomenclature-up-to-date/prokariotic-nomenclature-up-to-date.html) [10]. Members of *Lactobacillus* are gram-positive, non-motile, anaerobic, lactic-acid-producing bacilli that are divided into three fermentation groups: A) obligately homofermentative, B) facultatively heterofermentative, and C) obligately heterofermentative [4]. *L. rhamnosus* resides in fermentation group B and is distinct from the three major *Lactobacillus* phylogenetic groups based on 16S rRNA gene sequence (*L. delbrueckii*, *L. reuteri*, and *L. salivarius* groups) [4]. *L. rhamnosus* strains L31, L34, and L35 are phylogenetically similar to *L. rhamnosus* GG and maintain a distinctive 16S rRNA gene-based phyologeny from the three major *Lactobacillus* groups (Figure 1). The basic characteristics of *L. rhamnosus* L31, L34, and L35 are summarized in Table 1.

**Table 1:** Classification and general features of *L. rhamnosus* strains L31, L34, and L35 according to the MIGS recommendation

**MIGS ID**	**Property**	**Term**	**Evidence code^a^**
	Classification	Domain *Bacteria*	TAS [12]
		Phylum *Firmicutes*	TAS [13-15]
		Class *Bacillus*	TAS [16-18]
		Order *Lactobacillales*	TAS [19,20]
		Family *Lactobacillaceae*	TAS [16,21]
		Genus *Lactobacillus*	TAS [16,22-26]
		Species *Lactobacillus rhamnosus*	TAS [27]
		Strains L31, L34, and L35	IDA
	Gram stain	Positive	IDA
	Cell shape	Rod-shaped	IDA
	Motility	Non-motile	NAS
	Sporulation	Non-sporulating	NAS
	Temperature range	Mesophile	NAS
	Optimum temperature	37°C	IDA
	Carbon source	Glucose	NAS
	Energy source	Lactose, glucose and other sugars	NAS
**MIGS-6**	Habitat	Human GI Tract	NAS
**MIGS-22**	Oxygen	Facultative anaerobes	IDA
**MIGS-15**	Biotic relationship	Symbiotic relationship	NAS
**MIGS-14**	Pathogenicity	Nonpathogenic; potential probiotic	IDA
	Biosafety level	1	NAS
	Isolation	Infant Feces	IDA
**MIGS-4**	Geographic location	Bangkok, Thailand	IDA
**MIGS-5**	Sample collection time	Not reported	
**MIGS-4.1**	Latitude	13° 45’N	IDA
**MIGS-4.2**	Longitude	100° 35’E	IDA
**MIGS-4.4**	Altitude	Not reported	NAS

a) Evidence codes – IDA: Inferred from Direct Assay; TAS: Traceable Author Statement (i.e., a direct report exists in the literature); NAS: Non-traceable Author Statement (i.e., not directly observed for the living, isolated sample, but based on a generally accepted property for the species, or anecdotal evidence). These evidence codes are from the Gene Ontology project [28].

**Figure 2:**
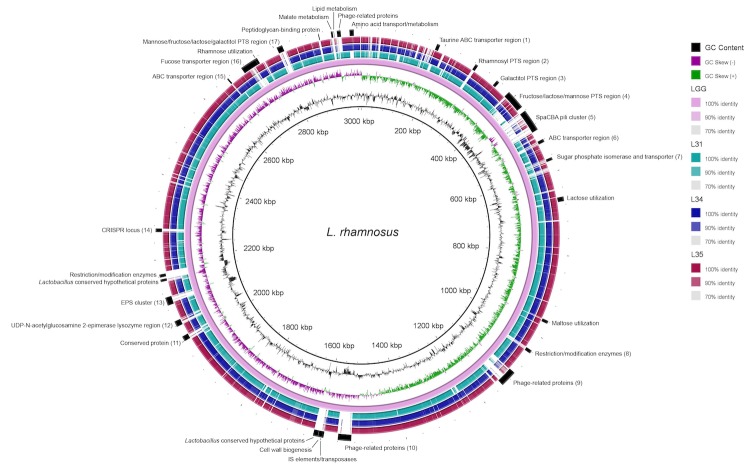
Colony morphology and Gram stains of *L. rhamnosus* strains L31, L34, and L35. *L. rhamnosus* strains were cultured anaerobically on MRS agar at 37°C for 48 hr. Gram stains were carried out using standard methods, and images were taken under oil emersion at 100× magnification.

The colony and Gram stain morphology of *L. rhamnosus* strains L31, L34, and L35 are each depicted in Figure 2. Supernatants from *L. rhamnosus* L34 and L35, both isolated from the same 40 day old female, suppress LPS-induced TNF-α production by THP-1 cells [9] and *C. difficile*-induced IL-8 production by HT-29 cells [29]. Similarly, strain L31, isolated from a 39 day old female, suppresses LPS-induced TNF-α production by THP-1 cells, however does not suppress *C. difficile*-induced IL-8 production by HT-29 cells [29]. All three strains are resistant to two drugs commonly used to treat *C. difficile* infection in humans, vancomycin and metronidazole (MIC90 >256µg/mL for each), but are susceptible to low concentrations (MIC90 = 2µg/mL) of the newest antibiotic targeting *C. difficile*, fidaxomicin. These strain-specific characteristics suggest *L. rhamnosus* L34 and L35 are potential probiotic candidates for either preventing or treating *C. difficile* disease.

## Genome sequencing information

### Genome project history

*L. rhamnosus* strains L31, L34, and L35 were selected for sequencing based on the properties described above. The draft genome sequence for each strain was finished in October 2012. The Whole Genome Shotgun projects for *L. rhamnosus* L31, L34, and L35 have been deposited at DDBJ/EMBL/GenBank under the accession numbers AYTQ00000000, AYTR00000000, and AYTP0000000, respectively. The versions described in this paper are AYTQ01000000, AYTR01000000, and AYTP0100000, respectively. The genome projects for L31, L34, and L35 are listed in the Genome OnLine Database (GOLD) [30] as projects Gi0036900, Gi0036903, and Gi0036905, respectively. Genome sequencing and assembly was completed at Baylor College of Medicine’s Human Genome Sequencing Center (BCM-HGSC). Automatic annotation was performed using the DOE-JGI Microbial Annotation Pipeline (DOE-JGI MAP). Table 2 shows the project information and its association with MIGS version 2.0 compliance [31].

**Table 2:** Project information

MIGS ID		**L31**	**L34**	**L35**
MIGS ID	Property	Term	Term	Term
MIGS-31	Finishing quality	Standard Draft	Standard Draft	Standard Draft
MIGS-28	Libraries used	8 kb, mate paired library	8 kb, mate paired library	8 kb, mate paired library
MIGS-29	Sequencing platforms	454 GS FLX	454 GS FLX	454 GS FLX
MIGS-31.2	Fold coverage	23×	29×	26×
MIGS-30	Assemblers	Newbler v2.5.3	Newbler v2.5.3	Newbler v2.5.3
MIGS-32	Gene calling method	Prodigal	Prodigal	Prodigal
	Genome Database release	March 1, 2014	March 1, 2014	March 1, 2014
	GenBank ID	AYTQ00000000	AYTR00000000	AYTP00000000
	GenBank Date of Release	March 1, 2014	March 1, 2014	March 1, 2014
	GOLD ID	Gi0036900	Gi0036903	Gi0036905
	Project relevance	Potential probiotic	Potential probiotic	Potential probiotic

### Growth conditions and DNA isolation

*L. rhamnosus* strains L31, L34, and L35 were routinely cultured in an anaerobic chamber (Concept Plus, Ruskinn Technology, UK) (10% CO_2_, 10% H_2_, and 80% N_2_) for 24-48 h at 37^°^C in de Man, Rogosa, Sharpe (MRS) medium (Oxoid, England). For genomic DNA isolation, cultures were adjusted to an OD_600_ of 0.1 and incubated anaerobically at 37°C for 8 h. Bacterial pellets were collected by centrifugation and the DNA was extracted using QIAGEN Genomic-tip100/G columns (Qiagen, Germany) according to the manufacturer’s instructions. DNA quality was analyzed by agarose gel electrophoresis, and concentrations were determined by fluorescence using the Qubit™ DNA Assay (Life Technologies, USA).

### Genome sequencing and assembly

The genomes of *L. rhamnosus* strains L31, L34, and L35 were sequenced at the BCM-HGSC, USA on a Roche 454 GS FLX sequencing platform. A fragment sequencing approach was implemented using 8 kb libraries generated by long insert mate paired construction, as detailed in the Human Microbiome Project Reference Genome Project protocol [32] to about 23× (254,342 reads), 29× (283,036 reads), and 26× (249,176 reads) sequence depth coverage, respectively, with an estimated read alignment error rate of 0.84%. The sequence data were assembled using the Newbler assembler version 2.5.3. The final assemblies resulted in 67 (L31), 51 (L34), and 51 (L35) contigs generating corresponding genome sizes of 2.8, 2.9, and 2.9 Mb in 3, 3, and 4 scaffolds.

### Genome annotation

Open Reading Frames (ORFs) were predicted using Prodigal [33,34] as part of the Oak Ridge National Laboratory genome annotation pipeline, followed by a round of manual curation using the JGI GenePRIMP pipeline [35]. The predicted protein coding sequences (CDSs) were translated and searched against the National Center for Biotechnology Information (NCBI) non-redundant database, UniProt, TIGRFam, Pfam, PRIAM, KEGG, COG, and InterPro databases [35]. These data sources were combined to assert a product description for each predicted protein. Additional gene prediction analysis and manual functional annotation was performed with the Integrated Microbial Genomes Expert Review (IMG-ER) platform [36]. Non-coding genes and miscellaneous features were predicted using tRNAscan-SE [37], RNAMMer [38], Rfam [39], TMHMM [40], and signalP [41].

## Genome properties

The properties and statistics for the three *L. rhamnosus* genomes are summarized in Table 3. The distribution of genes into COG functional categories for each genome is detailed in Table 4. The *L. rhamnosus* L31 genome was assembled into 67 contigs (ranging from 551 – 290,053 bp) forming one presumptive circular chromosome of 2,826,754 base pairs (46.73% GC content). A total of 2,749 ORFs were predicted: 2,687 are protein-coding genes, and 62 are RNA genes. A total of 2,173 (79.05%) protein-coding genes were assigned a putative function. The L34 genome was assembled into 51 contigs (ranging from 288 – 237,520 bp) forming a presumptive single circular chromosome of 2,937,717 base pairs (46.81% GC content). A total of 2,845 ORFs were predicted: 2,774 are protein-coding genes, and 71 are RNA genes. A total of 2,216 (77.89%) protein coding genes were assigned a putative function. Finally, the L35 genome was assembled into 51 contigs (687 – 226,797 bp) forming one presumptive chromosome of 2,937,403 base pairs (46.81%). A total of 2,842 ORFs were predicted: 2,772 are protein-coding genes, and 70 are RNA genes. A total of 2,217 (78.01%) protein coding genes were assigned a putative function.

**Table 3:** Nucleotide content and gene count level of the genomes

	**L31**		**L34**		**L35**	
**Attribute**	**Value**	%age^a^	**Value**	%age^a^	**Value**	%age^a^
Genome Size (bp)	2,826,754	100	2,937,717	100	2,937,403	100
DNA G+C content (bp)	1,320,949	46.73	1,375,266	46.81	1,375,134	46.81
DNA coding region (bp)	2,422,731	85.71	2,519,202	85.75	2,517,453	85.70
Total genes	2,749	100	2,854	100	2,842	100
RNA genes	62	2.26	71	2.50	70	2.46
Protein-coding genes	2,687	97.74	2,774	97.50	2,772	97.54
Genes with functional prediction	2,173	79.05	2,216	77.89	2,217	78.01
Genes in paralog clusters	1,818	66.13	1,898	66.71	1,869	65.76
Genes assigned to COGs	2,121	77.16	2,150	75.57	2,151	75.69
Genes assigned to KOGs	886	32.23	913	32.09	914	32.16
Genes assigned to Pfam	2,209	80.36	2,250	79.09	2,254	79.31
Genes assigned to TIGRfam	880	31.01	893	31.39	892	31.39
Genes with signal peptides	138	5.02	139	4.89	138	4.86
Genes with transmembrane helices	813	29.57	835	29.35	834	29.35

a) The total is based on either the size of the genome in base pairs or the total number of protein coding genes in the annotated genome.

**Table 4:** Number of genes associated with the 25 general COG functional categories

	**L31**		**L34**		**L35**		
**Code**	**Value**	**%age^a^**	**Value**	**%age^a^**	**Value**	**%age^a^**	**Description**
J	150	6.56	150	6.49	150	6.48	Translation
A	-	-	-	-	-	-	RNA processing and modification
K	203	8.88	206	8.91	207	8.95	Transcription
L	123	5.38	132	5.71	134	5.79	Replication, recombination and repair
B	-	-	-	-	-	-	Chromatin structure and dynamics
D	33	1.44	29	1.25	29	1.25	Cell cycle control, mitosis and meiosis
Y	-	-	-	-	-	-	Nuclear structure
V	75	3.28	79	3.42	79	3.41	Defense mechanisms
T	82	3.59	84	3.63	85	3.67	Signal transduction mechanisms
M	129	5.65	128	5.54	127	5.49	Cell wall/membrane biogenesis
N	9	0.39	8	0.35	8	0.35	Cell motility
Z	-	-	-	-	-	-	Cytoskeleton
W	-	-	-	-	-	-	Extracellular structures
U	28	1.23	23	0.99	23	0.99	Intracellular trafficking and secretion
O	59	2.58	59	2.55	59	2.5	Posttranslational modification, protein turnover, chaperones
C	90	3.94	87	3.76	88	3.80	Energy production and conversion
G	303	13.26	315	13.62	315	13.61	Carbohydrate transport and metabolism
E	183	8.01	178	7.70	177	7.65	Amino acid transport and metabolism
F	87	3.81	85	3.68	85	3.67	Nucleotide transport and metabolism
H	58	2.54	60	2.60	60	2.59	Coenzyme transport and metabolism
I	55	2.41	57	2.47	57	2.46	Lipid transport and metabolism
P	96	4.20	95	4.11	95	4.11	Inorganic ion transport and metabolism
Q	21	0.92	21	0.91	21	0.91	Secondary metabolites biosynthesis, transport and catabolism
R	285	12.47	292	12.63	291	12.58	General function prediction only
S	216	9.45	224	9.69	224	9.68	Function unknown
-	628	22.84	695	24.43	691	24.31	Not in COGs

a) The total is based on the total number of protein coding genes in the annotated genome.

## Comparison with *Lactobacillus rhamnosus* strain GG

**Figure 3:**
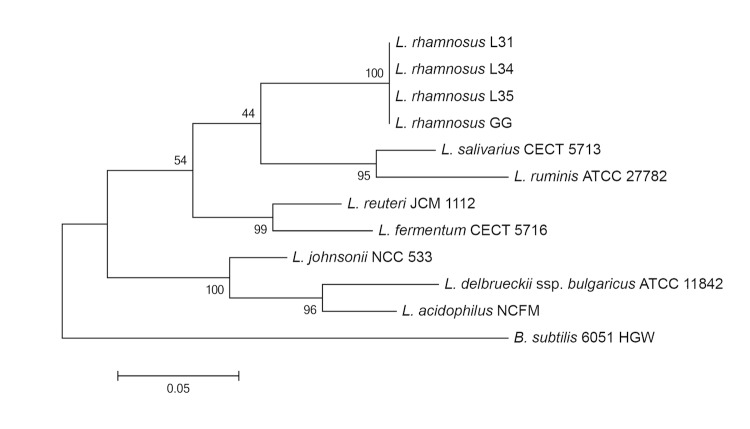
Circular representation of 3 draft *L. rhamnosus* genomes compared against *L. rhamnosus* strain GG (NC_ 013198). The innermost rings show GC content (black) and GC skew (purple/green). The remaining rings show BLASTn results of each genome against *L. rhamnosus* GG with results rendered using the BRIG program [48]. Relative shading density (from darker to lighter) within each circle represents levels of nucleotide homology. Blank regions represent absent genetic regions. Genetic regions of interest are annotated on the outermost ring. Numbered elements (1-17) represent the previously identified variable chromosomal regions of *L. rhamnosus* GG [47].

The beneficial effects of human-intestinal derived *L. rhamnosus* GG have been studied for two decades [42-45] and its complete genome is available in NCBI [46]. We have compared the draft genome sequences of the potential probiotic *L. rhamnosus* strains L31, L34, and L35 to *L. rhamnosus* GG. The *L. rhamnosus* GG genome (3,010,111 bp, 46.69% GC content) is slightly larger than the new genomes presented here, and has approximately the same GC content (Table 3). In a recent comparative genomics study of 100 *L. rhamnosus* strains, Douillard, *et al*. [47] delineated seventeen variable chromosomal regions of *L. rhamnosus* strain GG (annotated in Figure 3), and the majority of these regions are absent or incomplete in the genomes of strains L31, L34, and L35 (Figure 3), notably the *spaCBA* pili gene cluster required for mucus adhesion [46]. The galactitol PTS region important for dulcitol utilization, a trait that typically belongs to *L. rhamnosus* isolates adapted to the intestinal tract [47], is conserved in L31, L34, and L35. Similar to *L. rhamnosus* GG, L31, L34, and L35 each contain genes annotated as L-lactate dehydrogenase (*ldhL*) and D-lactate dehydrogenase (*ldhD*) important for synthesizing L-lactate and D-lactate from pyruvate, respectively [49]. *L. rhamnosus* GG is unable to metabolize either maltose due to an inserted gene between the maltose-specific transport genes and hydrolase, or lactose because of a 38 bp N-terminal truncation in *lacT* and a disrupted *lacG* [47,50]. Strains L31, L34, and L35 all have an intact maltose locus and carry non-mutated copies of *lacT* and *lacG* (locations indicated on Figure 3), and therefore are predicted to utilize both maltose and lactose.

In line with the anti-inflammatory phenotypic differences already noted [9,29], differences in genomic features between *L. rhamnosus* L31 and the two isolates, L34 and L35, can also be made relative to strain GG. The taurine transport system deemed important for bile resistance as well as the *fucU*, *fucI*, *fcsR*, and α-L-fucosidase genes required for metabolizing fucosylated compounds present in gastrointestinal environments are found in L34 and L35 genomes, but not in L31. *L. rhamnosus* GG, despite belonging to a species known for rhamnose utilization, possesses an altered rhamnose locus and cannot utilize rhamnose [46]. *L. rhamnosus* L31 contains an intact rhamnose locus, while this locus in strains L34 and L35 looks similarly disrupted to that of strain GG. It is also noteworthy that *L. rhamnosus* L31 contains an iron-transport and a general secretion system not present in strains L34, L35, or GG.

## Conclusion

Here we have presented the draft genomes of three potential probiotic strains of *L. rhamnosus*: L31, L34, and L35. Brief genome comparisons indicate that strains L34 and L35 are most similar to *L. rhamnosus* GG, while L31 contains marked differences suggesting it may have originated from a slightly different ecological niche [47]. *L. rhamnosus* L34 and L35 were isolated from the same host based on initial distinguishing colony morphology [9], however current colony morphology for these strains is not unique (Figure 2) and comparison of the draft genomes suggests the two genomes are nearly identical and similarly distinct from L31. It is possible that L34 and L35 may represent isolates of the same strain. Future studies will combine functional data with genomics, which is a powerful method for not only validating probiotic features of beneficial microbes, but also for learning about the environmental adaptations that have favored their mutual relationship with human hosts.
